# Senescence in Primary Rat Astrocytes Induces Loss of the Mitochondrial Membrane Potential and Alters Mitochondrial Dynamics in Cortical Neurons

**DOI:** 10.3389/fnagi.2021.766306

**Published:** 2021-12-01

**Authors:** Sandra Lizbeth Morales-Rosales, Roberto Santín-Márquez, Pedro Posadas-Rodriguez, Ruth Rincon-Heredia, Teresa Montiel, Raúl Librado-Osorio, Armando Luna-López, Nadia Alejandra Rivero-Segura, Claudio Torres, Agustina Cano-Martínez, Alejandro Silva-Palacios, Paulina Cortés-Hernández, Julio Morán, Lourdes Massieu, Mina Konigsberg

**Affiliations:** ^1^Posgrado Biología Experimental, Universidad Autónoma Metropolitana, Mexico City, Mexico; ^2^Departamento de Ciencias de la Salud, Universidad Autónoma Metropolitana, Mexico City, Mexico; ^3^Instituto de Fisiología Celular, Universidad Nacional Autónoma de México, Mexico City, Mexico; ^4^Departamento de Investigación Básica, Instituto Nacional de Geriatría, Mexico City, Mexico; ^5^Department of Neurobiology and Anatomy, Drexel University College of Medicine, Philadelphia, PA, United States; ^6^Departamento de Fisiología, Instituto Nacional de Cardiología Ignacio Chávez, Mexico City, Mexico; ^7^Departamento de Biomedicina Cardiovascular, Instituto Nacional de Cardiología Ignacio Chávez, Mexico City, Mexico; ^8^Instituto Mexicano del Seguro Social, Centro de Investigación Biomédica de Oriente, Atlixco, Mexico

**Keywords:** astrocyte, cellular senescence, redox state, aging, mitochondria

## Abstract

The decline in brain function during aging is one of the most critical health problems nowadays. Although senescent astrocytes have been found in old-age brains and neurodegenerative diseases, their impact on the function of other cerebral cell types is unknown. The aim of this study was to evaluate the effect of senescent astrocytes on the mitochondrial function of a neuron. In order to evaluate neuronal susceptibility to a long and constant senescence-associated secretory phenotype (SASP) exposure, we developed a model by using cellular cocultures in transwell plates. Rat primary cortical astrocytes were seeded in transwell *inserts* and induced to premature senescence with hydrogen peroxide [stress-induced premature senescence (SIPS)]. Independently, primary rat cortical neurons were seeded at the *bottom* of transwells. After neuronal 6 days *in vitro* (DIV), the inserts with SIPS-astrocytes were placed in the chamber and cocultured with neurons for 6 more days. The neuronal viability, the redox state [reduced glutathione/oxidized glutathione (GSH/GSSG)], the mitochondrial morphology, and the proteins and membrane potential were determined. Our results showed that the neuronal mitochondria functionality was altered after being cocultured with senescent astrocytes. *In vivo*, we found that old animals had diminished mitochondrial oxidative phosphorylation (OXPHOS) proteins, redox state, and senescence markers as compared to young rats, suggesting effects of the senescent astrocytes similar to the ones we observed *in vitro*. Overall, these results indicate that the microenvironment generated by senescent astrocytes can affect neuronal mitochondria and physiology.

## Introduction

Cellular senescence is a central and influential hallmark of aging (López-Otín et al., 2013; Cohen and Torres, 2019), able to promote tissue remodeling and repair, but also capable of inducing cellular and tissue dysfunction. The accumulation of senescent cells during aging has been related to a significant increase in inflammation and tumorigenesis. On the other hand, benefits of the clearance of senescent cells have been reported (Baker et al., 2011; Hernandez-Segura et al., 2018; Triana-Martínez et al., 2019).

The senescent phenotype is characterized by cellular morphological changes: a chronic DNA damage response, activation of cyclin-dependent kinase inhibitors, antiapoptotic genes, an increase in metabolism, and endoplasmic reticulum stress (Antelo-Iglesias et al., 2021). In addition, senescent cells, through the senescence-associated secretory phenotype (SASP), secrete a complex mixture of factors, including proinflammatory cytokines and tissue remodelers, that may adversely affect neighboring cells and tissues with chronic inflammatory signals and oxidative stress (Rodier and Campisi, 2011; Yan et al., 2014; Van Eldik et al., 2016).

The central nervous system (CNS) undergoes numerous detrimental transformations with age such as mitochondrial dysfunction, oxidative stress, and chronic inflammation (Chakrabarti et al., 2011; Maciel-Barón et al., 2018b) and the presence of senescent cells has been associated with neurodegenerative diseases including Alzheimer’s disease (AD), Parkinson’s disease (PD), and HIV-dementia (Bhat et al., 2012; Chinta et al., 2015, 2018; Cohen et al., 2018; Guerrero et al., 2021).

Brain metabolic homeostasis depends on tight integration between neurons and glia. Glia maintains the proper function of the neuronal mitochondria ensuring ATP production, lipid biogenesis, reactive oxygen species (ROS) elimination, and calcium regulation. Substrates as lactate provided by astrocytes represent an important precursor to ATP production in neurons (Kirchhoff et al., 2001; Ricci et al., 2009; Bouzier-Sore and Pellerin, 2013; Jha and Morrison, 2018). In addition, astrocytes display an important role as modulators of oxidative stress in neurons, providing GSH and glutamine as a substrate for the glutamate production used in GSH *de novo* synthesis (Dringen et al., 2000; Vicente-Gutiérrez et al., 2021). Taken together, this evidence indicates that astrocytes are vital protectors for neurons and the functional dysfunction associated with senescence may have profound implications for CNS and the etiology of neurological disorders (Maciel-Barón et al., 2018b; Cohen and Torres, 2019). The senescent state brings with it important changes encompassing the loss of specific functions and the acquisition of certain characteristics, such as the SASP, which are associated with neuronal dysfunction leading to neuropathology. Astrocyte senescence might impact the neuron either by the secretions the senescent astrocytes produce (such as the SASP) or by the metabolites they stop transferring, which are essential for neuronal support, thus modifying the mitochondrial content and the redox state. Hence, the aim of this study was to evaluate the effects that senescent astrocytes could have on neuronal functionality, in particular, on the mitochondrial function and dynamics. Our results showed that the neuronal mitochondria functionality was altered after being cocultured with senescent astrocytes. *In vivo*, old animals showed diminished mitochondrial oxidative phosphorylation (OXPHOS) proteins, redox state, and senescence markers as compared to young rats, suggesting effects of the senescent astrocytes similar to the ones we observed *in vitro*.

## Materials and Methods

### Chemicals

All the chemicals and reagents were purchased from Sigma Chemical Corporation (St. Louis, MO, United States). Reagents obtained from other sources are detailed throughout the text.

### Animals

The primary astrocytes were obtained from 3 to 7 days- old neonatal Wistar rats (*Rattus norvegicus*), provided by the closed breeding colony of the Universidad Autónoma Metropolitana-Iztapalapa (UAM-I). The primary neurons were isolated from embryonic Wistar rats (17–18 days of gestation), provided by the Instituto de Fisiología Celular (IFC), UNAM.

For the Western blot (WB) and the redox state determinations, the rats were also provided by the closed breeding colony at UAM-I. Young (4 months old) and old (24 months old) male rats were used. The animals were housed five per cage in polycarbonate cages, in a 12-h light-dark cycle, and provided with a standard commercial rat diet (Harlan 2018S, United States) and water *ad libitum.* All the procedures with the animals were strictly carried out according to the National Institutes of Health Guide for the Care and Use of Laboratory Animals and the Principles of the Mexican Official Ethics Standard 062-ZOO-1999 and the Standard for the Disposal of Biological Waste (NOM-087-ECOL-1995). This study was approved by the Ethics Committee of the Division of Biological and Health Sciences, UAMI, with the dictum 1851.

### Astrocyte Primary Culture

Astrocytes were isolated as described previously (McCarthy and de Vellis, 1980) and modified by Maciel-Barón et al. (2018a). Dissected brains were submerged in cold-sterile phosphate buffered saline (PBS) with 2% of antibiotic-antimycotic (Ab-Am), brought to the laminar hood, and decanted in a Petri dish. The brains were washed three times with sterile PBS and the cortex was dissected, cut into small fragments, and resuspended in 5 ml of sterile PBS in a 15-ml tube. With a 5-ml micropipette, the suspension was strongly resuspended 15 times to separate the cells from the tissue. The suspension was centrifuged for 10 min at 3,500 rpm. The pellet was recovered and resuspended in 8 ml of an astrocyte culture media (ACM) containing neurobasal medium (Gibco, 21103-049, Grand Island, NY, United States), supplemented with 10% fetal bovine serum (FBS)/1% Ab-Am/1.1% glutamine. The suspension was filtered in a cell strainer (pore 100 μm) and incubated for 24 h at 37°C–5% carbon dioxide (CO_2_). After 24 h, the cells were washed with cold-sterile PBS to eliminate other cell types. Astrocytes were observable by optical microscope 1 or 2 weeks after the isolation. ACM was changed two times a week. When cultures exceed 75% of confluence, the cells were trypsinized and reseeded at normal density (10^4^ cells/cm^2^).

### Cortical Neuron Primary Culture

Cortical neurons were isolated as described by Brewer et al. (1993) with modifications (Gerónimo-Olvera et al., 2017). The cerebral cortex was dissected and cut into small pieces and incubated with 0.25% trypsin/10% Ethylenediaminetetraacetic acid (EDTA) solution at 37°C for 3 min. The digestion was stopped with a solution containing soybean-trypsin inhibitor and DNAse (0.52 and 0.08%, respectively). The cells were suspended in a neuronal culture media (NCM) containing neurobasal medium (Gibco, 21103-049, Grand Island, NY, United States), supplemented with 1% of B27 (Gibco, 17504-044), 1% of B27 minus antioxidants (Gibco, 10889038), 0.5 mM L-glutamine, 20 μg/ml gentamicin (Gibco, 15710-064). Neurons were seeded at a 2.2 × 10^5^ cells/cm^2^ density at the bottom of the transwell chambers. The transwell plates were precoated with poly-L-lysine (Sigma-Aldrich, P-1524, St. Louis, MO, United States). The cells were cultured at 37°C and 5% CO_2_ and half of the NCM was renewed twice a week. The experiments were carried out at 12 days *in vitro* (DIV).

### Astrocytes Senescence Induction

Astrocytes senescence was induced by hydrogen peroxide (H_2_O_2_) as we have previously described (Maciel-Barón et al., 2018a). Non-senescent astrocytes were used at early population doublings (PD) (*PD* < 5) to avoid replicative senescence. Astrocytes were trypsinized and seeded at normal density (1 × 10^4^ cells/cm^2^) on the transwell inserts (0.4 μm membrane pore) (Corning Life Sciences, Acton, MA, United States) previously humidified with ACM overnight. After 3 days in culture, the astrocytes were incubated for 2 h with 75 μM H_2_O_2_ dissolved in the ACM at 37°C and 5% CO_2_. H_2_O_2_ was removed by changing the medium and the cells were allowed to recover fresh ACM for 2 more days before further measurements.

### Senescence-Associated-β-Galactosidase (SA-β-Gal) Staining

The senescence-associated-β-galactosidase activity was determined as previously described (Dimri et al., 1995). The cells were fixed with 3% formaldehyde. Three wells were stained 2, 4, 6, and 8 days after the induction of senescence and the number of SA-β-Gal-positive cells was determined by counting at least 100 cells per well under a microscope. The number of SA-β-Gal-positive cells is reported as the percentage of the total scored cells.

### Neuron and Astrocytes Coculture

**FIGURE 1:**
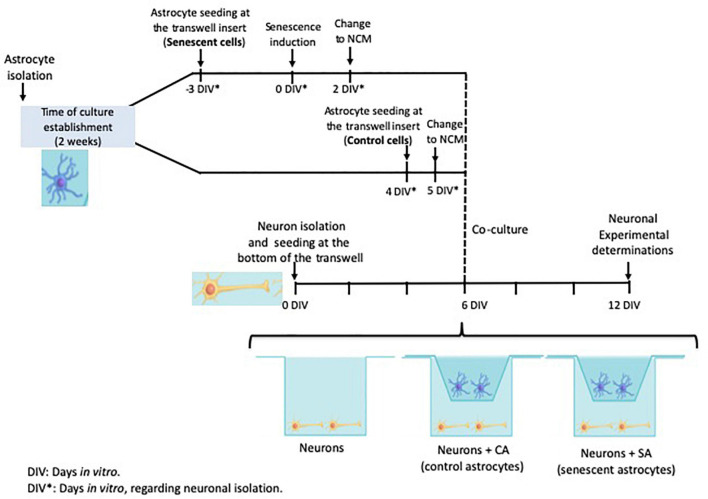
Cocultures experimental design. The timeline of each primary culture and the times selected to assemble the coculture are shown. Neuron isolation was used as the reference for the days *in vitro* (DIV). The neurons were seeded at the transwell bottom and the astrocytes were seeded at the transwell insert. The astrocytes were isolated on the same day and then split into the control and senescent groups.

Neurons and astrocytes were isolated independently as described above and the cocultures were assembled in transwell chambers as described below and in Figure 1. The isolated astrocytes were divided in which half of them was used as control astrocytes (CA) and the other half was induced to senescence astrocytes (SA). Besides the neurons with control and senescent astrocytes, other neurons were also seeded alone as a control.

a. *Neurons*. Newly isolated neurons were seeded at the bottom of the transwell chamber. The neurons were kept in NCM for 12 DIV when the experiments were performed. Half of the NCM was renewed two times a week.
b. *Neurons + CA*. Newly isolated neurons were seeded at the bottom of the transwell chambers in NCM as described above. The neurons were allowed to mature and generate their processes for 6 days. The astrocytes were isolated and handled separately as explained above. Two days before the coculture assembly, the control astrocytes were seeded in the transwell inserts in ACM. After 24 h, the ACM was replaced with NCM and the astrocytes were allowed to adapt for another 24 h. The coculture was performed on the six DIV for the neurons, when the transwell inserts, containing the CA, were placed over the wells in which neurons were previously seeded.
c. *Neurons + SA*. This coculture was carried out similarly to the previous one, except that the astrocytes were seeded in the transwell inserts 9 days before the coculture. After 3 days, senescence was induced as described above and the astrocytes were maintained in ACM for 2 days. After that time, the medium was changed to NCM. After 6 days of senescence induction, the transwell inserts containing senescent astrocytes were placed over the wells in which neurons were previously seeded.

The cocultures were maintained for 6 days (corresponding to 12 DIV for the neurons) and half of the NCM was renewed twice a week.

### Mitochondrial Membrane Potential Assay

After 6 days in coculture, corresponding to neurons 12 DIV, the mitochondrial membrane potential was assayed by using the cationic colorant JC1 (Thermo Fisher Scientific, MA, United States) (Rivero-Segura et al., 2019). For this assay, the newly isolated neurons were cultured in coverslips and laid inside the bottom of the transwell at day 0 and the normal coculture procedure was performed. At 12 DIV, the neurons were incubated with 100 nM JC-1 diluted in NCM and incubated for 30 min at 37°C in the dark. After that time, each coverslip was immersed into Locke’s solution [sodium chloride (NaCl) 154 mM, potassium chloride (KCl) 5.6 mM, sodium bicarbonate (NaHCO_3_) 3.6 mM, calcium chloride (CaCl_2_) 2.6 mM, N-(2-Hydroxyethyl)piperazine-N′-(2-ethanesulfonic acid), 4-(2-Hydroxyethyl)piperazine-1-ethanesulfonic acid (HEPES) 5 mM, and glucose 5.6 mM, pH 7.4] and observed in a confocal microscope (Leica TCS-SPS) by using the argon laser (488 nm excitation wavelength). Signals obtained at 590 nm for polarized mitochondrion and 525 nm for depolarized mitochondrion were quantified with the ImageJ software (Rasband, W.S., ImageJ, U. S. National Institutes of Health, Bethesda, MD, United States^1^) and the 590/525 nm ratio was calculated.

### Viability Assay

At 12 DIV, the cell survival fluorescent markers for live and dead cells calcein-AM 2 μM and ethidium homodimer 1 μm were added to the cultured neurons for 30 min. After that time, the cells were washed with Locke’s solution and images were obtained by using confocal microscopy (FV1000; Olympus, China) motorized FV10ASW 2.1 with Ar 488 laser [for fluorescein (FITC)] and Ar 596 nm (for ethidium) and images from the different treatments were captured.

### Mitochondrial Mass Analysis

JC1 images corresponding to active mitochondria (J-aggregates, 590 nm) were processed according to Merrill et al. (2017) and Baulies et al. (2018) with few modifications. Images from J-aggregates (590 nm) from each condition were processed with the rolling ball algorithm to subtract the background with Fiji.^2^ Then, the color map *Fire* was used to identify different shades of the same color in the 8-bit image. Consecutively, the images were converted to monochrome images with the binary tool, and the threshold was adjusted to black and white (mitochondrial mask) for further analysis. Finally, the mitochondrial mass was analyzed with the particle analyzer tool from Fiji with the size of the following parameters (pixel units) = 0.095-Infinity and circularity = 0.00--1.00. The results were plotted in GraphPad Software version 5.0 for Windows (La Jolla, CA, United States^3^)

### Mitochondrial Network

After 6 days in coculture, corresponding to the neuron cultured during 12 DIV, the mitochondrial network morphology was assayed by using MitoTracker Red (cat M7512, Thermo Fisher Scientific, MA, United States). For this assay, the neurons were also cultured in coverslips inside the bottom of the transwell as described above. At 12 DIV, the neurons were incubated in the dark for 30 min at 37°C with 100 nM MitoTracker Red diluted in NCM. After that time, each coverslip was immersed into Locke’s solution (as mentioned before) and observed live in a confocal microscope (Leica TCS-SPS) by using 543 nm as excitation wavelength and 599 nm as emission wavelength. The mitochondrial morphology was analyzed with the ImageJ software.

Mitochondrial network morphology was evaluated by classifying the neurons into one of three mitochondrial morphologies: tubular, fragmented, or intermediate. The length/width ratio of stained mitochondria was used to classify cells into the corresponding group. The cells were scored as having “tubular” mitochondria when distinct mitochondrial tubules were observed with length/width >20. Regularly, these tubules traversed at least one-fifth of the cell with length/width 25–100 and few tubules were present per cell, even if they coexisted with other types of mitochondrial morphologies. When the cells had only round mitochondria with length/width < 5, they were scored as fragmented. The cells that did not fall into any of the previous categories were scored as “intermediate” (short mitochondria with length/width mostly between 5 and 10 and no distinct longer tubules in the cell).

### Western Blot Assay

*a. Neurons*. At 12 DIV, the neurons were trypsinized and resuspended in lysis buffer mammalian protein extraction reagent (M-PER) (Pierce Chemical, Rockford, IL, United States) supplemented with protease inhibitors (complete; Roche Applied Science, Indianapolis, IN, United States), 1 mM phenylmethylsulfonyl fluoride (PMSF), and 0.1 mM dithiothreitol (DTT).

*b. Cerebral cortex (Cx)*. The Cx was isolated after the euthanization of the rats and stored at –70°C until used. A total of 100 mg of each dissected tissue were mechanically homogenized in lysis buffer.

Neuronal or tissue homogenates were incubated at 4°C for 5–10 min and centrifuged at 14,000 g and 4°C for 20 min. The protein concentration was determined in the supernatants by using a commercial Bradford reagent (Bio-Rad Laboratories, Hercules, CA, United States). To prepare the samples for the sodium dodecyl sulfate-polyacrylamide gel electrophoresis (SDS-PAGE), we used the Laemmli buffer and the reducing agent 2-mercaptoethanol (Bio-Rad Laboratories, Hercules, CA, United States). Mixed samples were heated at 90–100°C for 5 min and placed on ice for 5 min. A total of 30 μg of the protein were loaded on the gel. Cell lysates were separated on 12% SDS-PAGE and transferred to polyvinylidene difluoride membranes (Invitrogen, Waltham, MA, United States) by using a semidry blotting process (Trans-Blot Turbo System; Bio-Rad Laboratories, Hercules, CA, United States). The membranes were then blocked with 10% bovine serum albumin (BSA) in PBS for 1 h, followed by excess removal with TRis buffered saline (TBS)-Tween rinses, and then probed with primary antibodies specific to anti-Drp1 (1:500, SC32898), anti-Fis1 (1:500, ab50838), anti-Opa1 (1:500, SC-393296), anti-Mfn2 (1:1,000, ab71498), anti-GLB (1:1,000, ab55177), anti-p16 (1:750, MAB4133), anti-p38 (1:1,000, ab31828) anti-p105 (1:1,000, SC-293141), anti-OXPHOS (1:4,000, ab110413), and anti-actin (1:1,000, SC47778). Antibodies were incubated overnight at 2–8°C; the membranes were washed three times with TBS-Tween and incubated for 2 h with the secondary antibody antirabbit (SC2357) or antimouse (SC358914) at a dilution of 1:1,000. The membranes were then washed three times with TBS-Tween horseradish peroxidase-conjugated secondary antibodies (Pierce, Rockford, IL, United States) for 1 h. After the three washes, blots were developed by using a commercial chemiluminescent reagent (SuperSignal Pierce, Rockford, IL, United States). Signal development was carried out in the WB and Chemiluminescence Imaging System (FUSION FX Vilber Lourmat, Vilber smart imaging).

### Redox State (Reduced Glutathione/Oxidized Glutathione Ratio)

The GSH and GSSG content was determined as described by Posadas-Rodríguez et al. (2020) with modifications. The treated cells were trypsinized and homogenized in hydrochloric acid (HCl)/Bathophenanthrolinedisulfonic acid disodium salt hydrate (BPDS) (HCl 10%/BPDS 1 mM), while the Cx (100 mg) was mechanically homogenized in 1 ml of hydrochloric acid/BPDS. The homogenates were then centrifuged at 13,500 rpm at 4°C for 5 min. Supernatants were recovered and 100 μl from each sample were injected into the high performance liquid chromatography (HPLC) system. The 1525 Waters Binary Pump coupled to a UV/Vis 2489 (210 nm) was used. The stationary phase used was the ZORBAX Eclipse XDB-C18, 4:6 mm × 250 mm, 5 μm column with acetonitrile 1% and potassium phosphate monobasic buffer 99% (20 mM Potassium phosphate monobasic (KH_2_PO_4_); pH 2.7). An isocratic flow of 1 ml/min was used. The samples were analyzed by UV detection at 210 nm. The area under the curve was determined by using GSH and GSSG standards at different concentrations (10, 25, 50, 100, 200, and 400 μM).

### Statistical Analysis

Data are reported as the mean ± SD for at least three independent experiments by using cells from different donor animals. The ANOVA test was followed by the Tukey–Kramer variance analysis. *P* < 0.05 level of probability was used as a minimum criterion of significance.

## Results

### Neuronal Viability After Coculture With Senescence Astrocytes and Control Astrocytes

Neuron and astrocyte cocultures were performed as shown in Figure 1. Senescence induction in rat primary astrocytes was performed as we previously reported (Maciel-Barón et al., 2018a). To make sure that astrocytes were senescent before cocultures assays, a proliferation curve and the SA-β-Gal activity were determined (Supplementary Figure 1). Six days after the H_2_O_2_ treatment, 79.6% of astrocytes were senescent; therefore, this day was selected to plate astrocytes in coculture with neurons.

**FIGURE 2:**
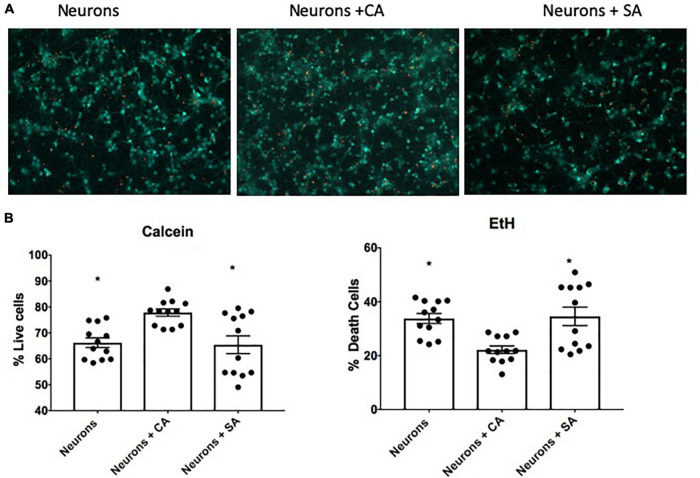
Neuronal viability. **(A)** Representative images of the live-death assay by using calcein and ethidium homodimer. Neuronal viability was assessed at 12 DIV after being alone or in coculture with senescent astrocytes (SA) and control astrocytes (CA). **(B)** Percentage of living (calcein-positive cells) and death (EtH-positive cells). Each bar represents the mean ± SD; *n* = 3 for each group. Significant differences were tested by the ANOVA and the Tukey–Kramer test, *p ≤ 0.05 vs. neurons + CA.

After 6 days in co-culture, the neuronal viability was evaluated. As shown in Figure 2, when the neurons were cocultured with CA, the percentage of living cells was 77.8%, which was significantly different from the neurons cultured alone (66.2%) and the neurons cocultured with SA (65.4%). The last two groups were not different between them. The difference in survival suggests that astrocytes improve neuronal endurance and that this beneficial effect was abrogated when the astrocytes became senescent.

### Mitochondrial Membrane Potential (ΔΨm) Decreases in Neurons Cocultured With Senescence Astrocytes and the Redox State Becomes More Oxidized

**FIGURE 3:**
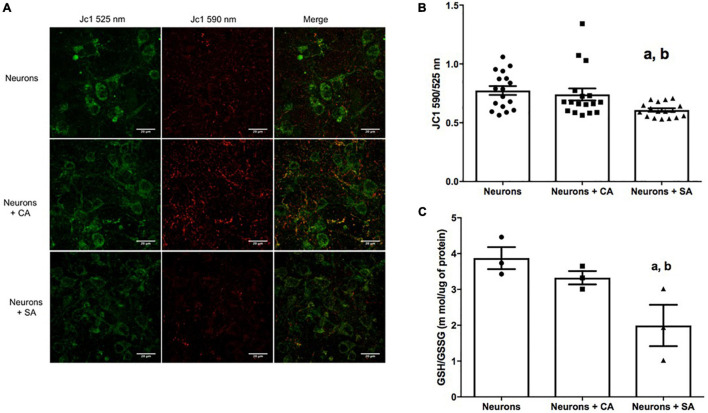
Senescent astrocytes decrease neuronal mitochondrial membrane potential (ΔΨm) and the redox state. **(A)** Neurons representative micrographs. ΔΨm was assessed by using the cationic colorant JC1 as described in materials and methods; the J-aggregates were determined at 590 nm (green) and the single molecules were determined at 525 nm (red). **(B)** 590/525 nm ratio quantification. **(C)** Reduced glutathione/oxidized glutathione (GSH/GSSG) ratio. Each bar represents the mean ± SE of three independent experiments for panels **(A,B)** and two experiments for panel **(C)**. Statistical significance with respect to a. *p* ≤ 0.05 vs. neurons and b. *p* ≤ 0.05 vs. neurons +CA, tested by the ANOVA and the Tukey–Kramer test.

mitochondrial neurons (ΔΨ) were evaluated with the cationic dye JC1. The coculture with senescent astrocytes significantly decreased the neuronal mitochondrial membrane potential as shown in Figure 3A and quantified in Figure 3B. The mitochondrial membrane potential value for the neurons was 0.608, which represents 21% less than neurons cultures alone (0.774) and 17% less than neurons + CA (0.741). Due to the loss of the ΔΨm, we evaluated the neuronal redox state by measuring the GSH/GSSG ratio in cellular lysates. Interestingly, the neurons that were cocultured with the SA (neurons + SA) showed a significantly more oxidized state 1.993, which represents 48% more than the neurons cultured alone (3.874) and 40% more than the neurons + CA (3.319) (Figure 3C).

### Mitochondrial Mass Decreases and the Mitochondrial Dynamics Are Altered in Neurons Cocultured With Senescence Astrocytes

**FIGURE 4:**
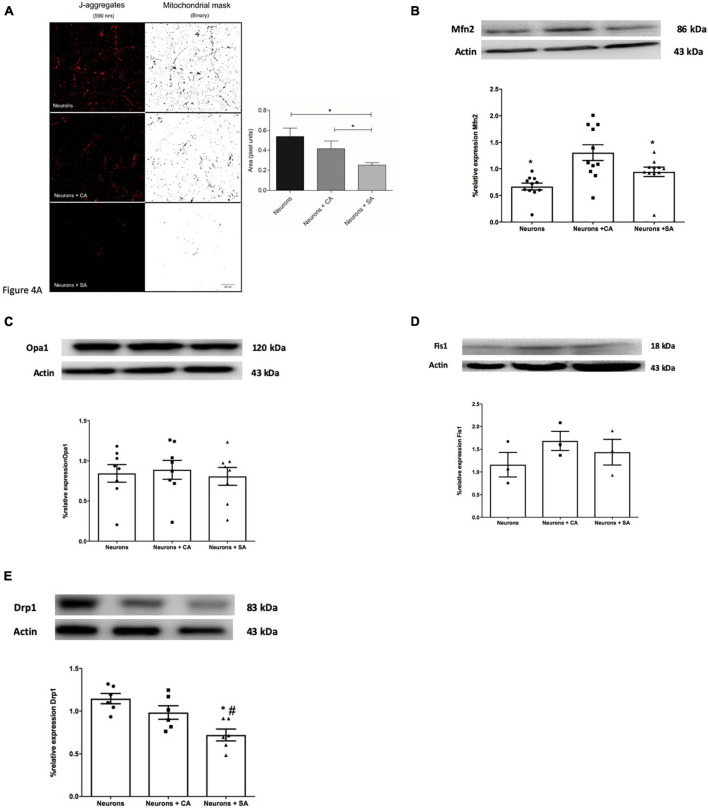
Senescent astrocytes decrease the mitochondrial mass in primary cultures of cortical neurons and change mitochondrial dynamics. **(A)** Images show the J-aggregates (590 nm) corresponding to active mitochondria and their mitochondrial mask processed with the binary filter. Mitochondria mass analysis, performed in Fiji software, shows a significant decrease in the mitochondrial area corresponding to the neurons exposed SA. Mitochondrial mass analysis is expressed in pixels area and each column represents the mean ± SD of at least five areas of four independent experiments. *p ≤ 0.05. Scale bar = 20 μm. **(B–E)** Mitochondrial dynamics-related proteins were evaluated by Western blot on neurons lysates as described in the methodology. Each bar represents the mean ± SE of three independent experiments. Statistical significance was tested by the ANOVA with respect to neurons group ^#^*p* ≤ 0.05 and neurons + CA group. *p ≤ 0.05. Representative images of the blot and densitometric analysis for **(B)** Mfn2, **(C)** total OPA1, **(D)** Fis1, and **(E)** Drp1.

The mitochondrial mass was evaluated by using the active mitochondria JC1 images (J-aggregates, 590 nm). Figure 4A shows that there is a decrease in mitochondrial mass in the neurons + SA, which was significantly different from that of the neurons alone and the neurons + CA.

The proteins that execute mitochondrial fusion (Opa1 and Mfn2) or fission (Fis1 and Drp1) were determined through the WB of neurons. Mfn2 significantly decreased 0.36 times in the neurons + SA and 0.94 times in the neurons alone compared with the neurons + CA (Figure 4B). No differences were determined for OPA1 and Fis1 (Figures 4C,D), but a significant decrease was observed in Drp1 in the neurons + SA compared to the other groups (Figure 4E).

The mitochondrial network morphology was evaluated by using MitoTracker Red staining and classifying the neurons into one of three basic mitochondrial morphologies: tubular, fragmented, or intermediate (Supplementary Figure 2A). Representative images are shown in Supplementary Figure 2B, where red squares emphasize the different mitochondrial morphologies found in the neurons. Cells were counted blindly and the results are shown in Supplementary Figure 2C. Almost 60% of the neurons alone or with SA were fragmented and <20% were tubular, while the neurons + CA showed predominantly an intermediate morphology (60%), 32% were fragmented, and <10% were tubular. These changes in mitochondrial morphology suggest that there might be alterations in fusion and fission events, which could be related to mitochondrial dysfunction.

### Senescence and Mitochondrial Marks in the Cerebral Cortex of Old and Young Rats

**FIGURE 5:**
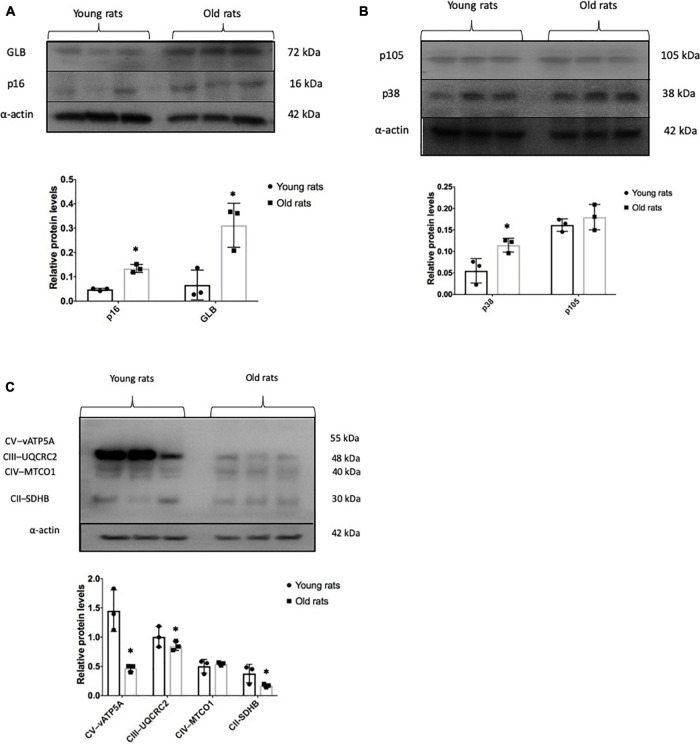
Senescent and mitochondrial marks in the old and young cerebral cortex. Representative Western blots and densitometric analysis of proteins isolated from young (4 months old) and old (24 months old) rats as described in the methodology. **(A)** Senescent marks: p16 and GLB. **(B)** Proteins related to senescence-associated secretory phenotype (SASP) production. p38 and p105. **(C)** Representative proteins in mitochondrial OXPHOS. Each bar represents the mean ± SE of three independent experiments. Statistical significance with respect to young rats ^∗^*p* ≤ 0.05.

**FIGURE 6:**
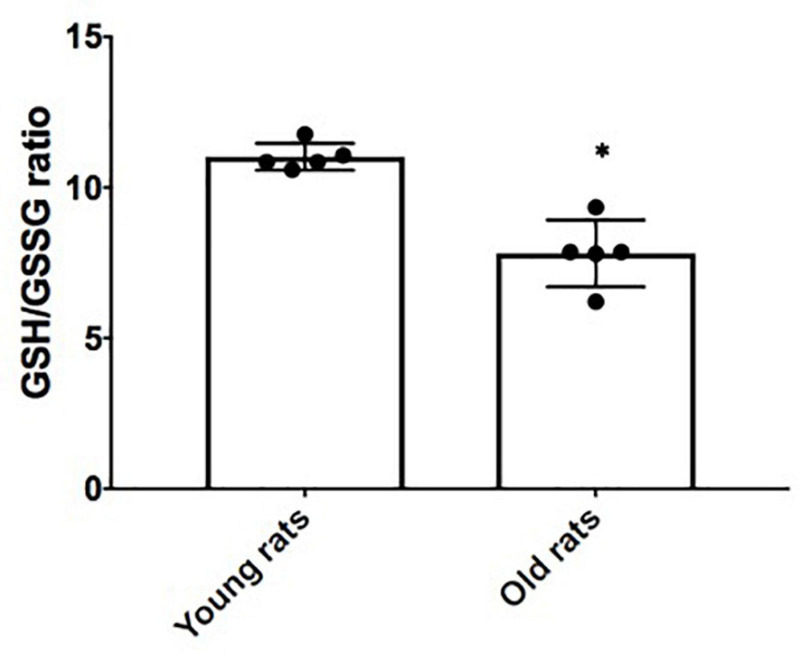
Redox state in the cerebral cortex (Cx) from young and old rats. Reduced glutathione/oxidized glutathione (GSH/GSSG) ratio was determined by using the proteins isolated from young (4 months old) and old (24 months old) rats as described in the methodology. Each bar represents the mean ± SE of three independent experiments. Statistical significance with respect to young rats ^∗^*p* ≤ 0.05.

In order to verify *in-vivo* alterations in cellular senescence and mitochondrial content during brain aging, we evaluated the status of several senescences and mitochondrial markers in the Cx from old and young rats. Beta-galactosidase (GLB) and p16 were evaluated as proteins related to the senescent phenotype. As illustrated in Figure 5A, they show a 4- and 2-fold increase, respectively, compared to young counterparts. Inflammation markers or SASP-related proteins such as p38 and p105 were determined. p38 augmented by 2-fold, but no significant difference was found in p105 between aged and young Cx (Figure 5B). Since we demonstrated in Figure 4A that there is a significant decrease in mitochondrial mass in the neurons + SA, we evaluated the OXPHOS components in the Cx from young and aged rats. Figure 5C shows that there is a significant decrease in the mitochondrial complexes, particularly in CV (3-fold), CIII (1.1-fold), and CII (2.17-fold). However, it was not possible to resolve CI in these conditions. Finally, the redox state was also evaluated in the Cx and as shown in Figure 6, we confirm the findings observed *in vitro*, with a significant decrease of 30% in the GSH/GSSG ratio in aged brains.

## Discussion

Senescent astrocytes have been defined as brain components associated with aging and AD (Bhat et al., 2012). Our group has characterized the SASP in rat astrocytes in response to oxidative stress and proteasome inhibition (Maciel-Barón et al., 2018a). Nevertheless, the number of studies related to cellular senescence within the CNS and the impact that senescent astrocytes have on the physiology and functionality of the neurons are limited.

Astrocytes comprise one of the most numerous glial cell populations and their function is key to several important processes such as synapse modulation, neurotransmitter and trophic factors regulation, mitochondrial substrates production, and others (Bouzier-Sore and Pellerin, 2013; Matias et al., 2019). Thus, any change in astrocytes functionality, such as those associated with the acquisition of the senescent phenotype, might have an important impact on brain functioning, particularly on neurons. A recent publication by using astrocyte and neuron cocultures found that treating senescent astrocytes with two different p53 isoforms modifies the SASP, confirming that SASP of the astrocytes is able to induce neurotoxicity (Turnquist et al., 2016). Moreover, it has also been reported that senescent astrocytes have altered glutamate transporters homeostasis, causing neuronal death (Limbad et al., 2020).

In this study, we analyzed the effects of SA on neuronal mitochondrial dysfunction by using a novel coculture model composed of astrocytes and neurons that are not in direct contact with each other, supporting a role for SA as modifiers of neuronal homeostasis. In this study, a higher percentage of living cells was found in the neurons that were cocultured with CA (neurons + CA), while the neurons cocultured with SA (neurons + SA), survived the same as the neurons alone (Figures 2A,B). However, with respect to the mitochondrial membrane potential, a decrease was only found in the neurons + SA. These results suggest two possible scenarios: in the first one, the SA might have stopped secreting factors required for the correct function of neurons, since the outcome observed in the first assay was similar in neurons cultured alone or neurons + SA. This is in line with this study mentioned before, where astrocytes lost their basal functions involved in glutamate reuptake, thus harming the neurons (Limbad et al., 2020). In the second scenario, the SASP secreted by the SA might have a detrimental influence on the functionality of neurons, since only neurons + SA decreased their mitochondrial potential. A similar effect was observed in cocultures performed by Turnquist et al. (2016), where the SA had a neurotoxic effect attributed to the SASP. Nevertheless, in previous studies, the cells were in direct contact with each other. The improvement observed in Figures 2A,B in the neurons + CA might be due to the contribution of nutrients that this type of glia provides to neurons, which, when senesce, cease to be supplemented as astrocytes become senescent (Pannese, 2021).

It is known that astrocytes are mainly glycolytic and synthesize lactate from pyruvate, which is released and taken up by neurons, then it is reoxidized to pyruvate to continue with oxidation in the tricarboxylic acid cycle, where energy is obtained through oxidative phosphorylation (Dienel, 2017; Takahashi, 2021). It has been observed that astrocytes have an age-related metabolic change from glycolytic metabolism to oxidative metabolism, carried out by the mitochondria (Jiang and Cadenas, 2014). Astrocyte senescence has also been reported to be accompanied by a general upregulation in mitochondrial metabolism (Cohen et al., 2017). Moreover, high metabolic activity is important for the development of SASP (Wiley and Campisi, 2016); so, in aged brains, astrocyte lactate input might no longer be supplied to the neurons. Interestingly, the percentage of dead cells that we observed in the neurons and neurons + SA was the same, but it was only a 10% decrease compared to the group of neurons + CA. Increasing the time in coculture might be an interesting proposal for future studies that could lead to gradual neuronal death as described during some types of neurodegeneration.

The proinflammatory components within the SASP have been considered as senescence markers of great relevance related to the chronic inflammation observed during aging or “inflammaging” (Franceschi et al., 2007), related to diverse chronic illnesses. It is known that the accumulation of senescent cells during aging contributes to an increase in neuroinflammation in older adults (Cohen and Torres, 2019). The SASP secreted by senescent cells contains a variety of proteins such as cytokines, interleukins, chemokines, proteases, growth factors, and degrading enzymes such as matrix metalloproteinases and insoluble extracellular matrix components (Perrott et al., 2017; Ritschka et al., 2017; Maciel-Barón et al., 2018a), which may be contributing to neuroinflammation.

Since inflammatory mediators are known to increase ROS and to alter mitochondrial metabolism (Missiroli et al., 2020), we determined if the SA could be affecting ROS generation and the redox state in neurons through the quantification of GSH/GSSG. A change in the cellular redox state induced by ROS increase could initiate and propagate chain reactions that damage important biomolecules, such as phospholipids, proteins, and DNA, leading to altered membrane properties, enzymes, and receptors inactivation, genomics instability, etc. (Chakrabarti et al., 2011). Our results showed that neurons + SA modified their redox state (Figure 3C), turning it into a more oxidized cellular environment, and the same result was confirmed in Cx from old rats. Another factor that could be affecting the redox state is the mitochondrial dysfunction itself since in the brain mitochondrial function is of great importance. In neurons, mitochondria are widely distributed in dendrites and axons, where their energy supply is key for chemical neurotransmission and cell maintenance (Mattson et al., 2008). Mitochondrial dysfunction has also been proposed as one of the hallmarks of aging (López-Otín et al., 2013); since during such process, the mitochondria lose functionality due to three possible causes: decreased electron transfer, increased internal membrane permeability to H^+^, and the decrease in ATP synthesis (Boveris and Navarro, 2008). On the other hand, it has been observed that during aging, there is an increase in point mutations and deletions in mitochondrial DNA, which is close to the ROS generation sites. This damage might, in turn, induce alterations in respiratory chain complexes, promoting mitochondrial dysfunction (Theurey and Pizzo, 2018).

Multiple alterations related to the mitochondria have been described in the brains of aged animals, such as changes in their morphology, either due to elongation or fragmentation, changes in the electron transport chain, and a depolarization of the mitochondrial membrane potential (Δψm) (LaFrance et al., 2005; Mattson and Arumugam, 2018). Our results with respect to the decrease in the mitochondrial membrane potential of neurons + SA correlate with the redox state oxidation and with the reports of Δψm affected by the respiratory complexes malfunction, which at the same time may increase ROS generation, affecting cellular functionality. Therefore, to complement this idea, it would be important to determine ATP synthesis in neurons + SA to confirm mitochondrial dysfunction.

It is essential for the cells to constantly regulate mitochondrial size, morphology, and number. The mitochondrial network is regulated by the two opposing processes of mitochondrial fusion and fission, which are carried out continuously in accordance with cellular needs. Fission is regulated by Drp1, a cytosolic protein that is recruited to mitochondria by different adapters such as Fis1, Mff, Mid49, and Mid51, while fusion is a sequential process that joins the external mitochondrial membrane by mitofusins 1 and 2 (Mfn1 Mfn2) and then the inner mitochondrial membrane by Opa1 (Cid-Castro et al., 2018), resulting in a tubular-shaped mitochondrial network. Our results are pioneers in relating the influence that senescent astrocytes have on the mitochondrial remodeling processes in neurons. Neurons + CA presented a mixture of tubular and fragmented mitochondrial morphology. In contrast, neurons alone and neurons + SA showed predominantly fragmented mitochondria, which, in turn, correlated with decreased Mfn2 (Cid-Castro et al., 2018), with mitochondrial dysfunction suggested by a decrease in the mitochondrial membrane potential and with decreased mitochondrial mass in neurons + SA. A decrease in the fission protein Drp1 was also observed in neurons + SA. It is known that Drp1 activity is regulated by a large number of post-translational modifications (Nakamura et al., 2010) that were not evaluated in our model and that could increase the mitochondrial fission activity even without increased Drp1. Mitochondrial morphology does not linearly correlate with the number of proteins that execute these events, as many of these proteins are not limiting and are regulated post-translationally. Unbalanced mitochondrial dynamics are known to cause structural mitochondrial disturbances and dysfunction. Failure of mitochondrial fission can lead to the accumulation of damaged and inactive mitochondria, which increase ROS production, while inadequate mitochondrial fusion results in mitochondrial fragmentation, leading to increased mitochondrial destruction (Adaniya et al., 2019). So, altered mitochondrial equilibrium between these two processes may contribute to neuronal dysfunction. It would be interesting to include in future studies real-time experiments of mitochondrial dynamics in the cocultures.

The results obtained *in vivo* confirm that the Cx from old rats had more senescence markers than the young animals such as p16 and GLB. This concurs with what has been reported in the brain of elderly people and patients with AD (Bhat et al., 2012). Yet, more experiments are needed to determine the types of brain cells that are senescent. Likewise, it was found that other markers that promote SASP were also increased, while the proteins related to mitochondrial OXPHOS decreased, therefore suggesting that a senescent microenvironment promoted by the astrocytes might affect the neuronal physiology and that a possible target are the neuronal mitochondria. However, more studies are needed to determine if the glia or the neurons are the prominent senescent cells during normal aging in order to develop the therapeutic strategy to follow before eliminating all the senescent cells in the brain.

## Conclusion

It is very important to understand how SA could modify neuronal homeostasis, in particular, mitochondrial function. In this case, the changes in neuronal functionality might be attributed both to the SASP secreted by SA and to the important substrates with which they normally supplement neurons that may be discontinued in SA. Unlike other experiments that use conditioned media or cocultures in direct interaction, this study simulated a scenario where the neurons microenvironment is modified by SA. Moreover, decreased mitochondrial OXPHOS proteins, redox state, and senescent markers were also found in the cortex of old animals, correlating with the *in-vitro* results. In this study, we present for the first time, to the best of our knowledge, the effect of astrocyte senescence on mitochondrial neurons, suggesting that the modulation of the brain microenvironment may be a potential target to prevent neuronal dysfunction during aging.

## Data Availability Statement

The original contributions presented in the study are included in the article/Supplementary Material, further inquiries can be directed to the corresponding author.

## Ethics Statement

The animal study was reviewed and approved by the Comisión Académica de ética de la División de Ciencias Biológicas y de la Salud, Universidad Autónoma Metropolitana, Unidad Iztapalapa.

## Author Contributions

SM-R was involved in the design of the study, generation, collection, and interpretation of the data and the manuscript writing. RS-M, PP-R, RR-H, TM, RL-O, AC-M, and AS-P assisted in the generation, collection, and interpretation of the data. CT, AL-L, NR-S, and PC-H were involved in the analysis and interpretation of the data. JM, LM, and MK were involved in the design of the study, analysis of the data, and revision of the manuscript. MK also supervised the investigation. All authors contributed to the article and approved the submitted version.

## Conflict of Interest

The authors declare that the research was conducted in the absence of any commercial or financial relationships that could be construed as a potential conflict of interest.

## Publisher’s Note

All claims expressed in this article are solely those of the authors and do not necessarily represent those of their affiliated organizations, or those of the publisher, the editors and the reviewers. Any product that may be evaluated in this article, or claim that may be made by its manufacturer, is not guaranteed or endorsed by the publisher.

## Footnotes

1
https://imagej.nih.gov/ij/

2
https://imagej.net/Fiji

3
www.graphpad.com

## Supplementary Material

Supplementary Figure 1 **(A)** Astrocytes proliferation. Cellular proliferation was determined every other day in hydrogen peroxide (H_2_O_2_) treated and untreated astrocytes. Each point represents the mean ± SD of three independent experiments. Statistical significance tested by the ANOVA and Tukey–Kramer test ^∗^*p* ≤ 0.05 with respect to control cells. **(B)** Senescence-associated-β-galactosidase (SA-β-Gal) staining. Representative images of H_2_O_2_ treated and untreated astrocytes. **(C)** SA-β-Gal-positive cells quantification. Each point represents the mean ± SD of three independent experiments. Statistical significance tested by the ANOVA and the Tukey–Kramer test ^∗^*p* ≤ 0.05 with respect to control cells.

Supplementary Figure 2 Mitochondrial morphology was evaluated by fluorescent microscopy by using MitoTracker red as described in the methodology. **(A)** Representative images of the three basic morphologies used for classification: tubular, fragmented, and intermediate. **(B)** Representative images observed in neurons, neurons +CA, and neurons +SA. **(C)** The percentage of evaluated morphologies in each condition. A total of 50 cells were blindly counted per treatment.

## References

[B1] Adaniya S. M. O-Uchi J. Cypress M. W. Kusakari Y. Jhun B. S. (2019). Posttranslational modifications of mitochondrial fission and fusion proteins in cardiac physiology and pathophysiology. *Am. J. Physiol. Cell Physiol.* 316 C583–C604. 30758993 10.1152/ajpcell.00523.2018 PMC6580160

[B2] Antelo-Iglesias L. Picallos-Rabina P. Estévez-Souto V. Da Silva-Álvarez S. Collado M. (2021). The role of cellular senescence in tissue repair and regeneration. *Mech. Ageing Dev.* 198:111528. 10.1016/j.mad.2021.111528 34181964

[B3] Baker D. J. Wijshake T. Tchkonia T. LeBrasseur N. K. Childs B. G. van de Sluis B. (2011). Clearance of p16Ink4a-positive senescent cells delays ageing-associated disorders. *Nature* 479 232–236. 10.1038/nature10600 22048312 PMC3468323

[B4] Baulies A. Montero J. Matías N. Insausti N. Terrones O. Basañez G. (2018). The 2-oxoglutarate carrier promotes liver cancer by sustaining mitochondrial GSH despite cholesterol loading. *Redox Biol.* 14 164–177. 10.1016/j.redox.2017.08.022 28942194 PMC5609874

[B5] Bhat R. Crowe E. P. Bitto A. Moh M. Katsetos C. D. Garcia F. U. (2012). Astrocyte senescence as a component of Alzheimer’s disease. *PLoS One* 7:e45069. 10.1371/journal.pone.0045069 22984612 PMC3440417

[B6] Bouzier-Sore A.-K. Pellerin L. (2013). Unraveling the complex metabolic nature of astrocytes. *Front. Cell. Neurosci.* 7:179. 10.3389/fncel.2013.00179 24130515 PMC3795301

[B7] Boveris A. Navarro A. (2008). Brain mitochondrial dysfunction in aging. *IUBMB Life* 60 308–314. 10.1002/iub.46 18421773

[B8] Brewer G. J. Torricelli J. R. Evege E. K. Price P. J. (1993). Optimized survival of hippocampal neurons in B27-supplemented Neurobasal, a new serum-free medium combination. *J. Neurosci. Res.* 35 567–576. 10.1002/jnr.490350513 8377226

[B9] Chakrabarti S. Munshi S. Banerjee K. Thakurta I. G. Sinha M. Bagh M. B. (2011). Mitochondrial dysfunction during brain aging: role of oxidative stress and modulation by antioxidant supplementation. *Aging Dis.* 2 242–256. 22396876 PMC3295058

[B10] Chinta S. J. Woods G. Demaria M. Rane A. Zou Y. McQuade A. (2018). Cellular senescence is induced by the environmental neurotoxin paraquat and contributes to neuropathology linked to Parkinson’s Disease. *Cell Rep.* 22 930–940. 10.1016/j.celrep.2017.12.092 29386135 PMC5806534

[B11] Chinta S. J. Woods G. Rane A. Demaria M. Campisi J. Andersen J. K. (2015). Cellular senescence and the aging brain. *Exp. Gerontol.* 68 3–7. 10.18632/aging.100871 25281806 PMC4382436

[B12] Cid-Castro C. Hernández-Espinosa D. R. Morán J. (2018). ROS as regulators of mitochondrial dynamics in neurons. *Cell. Mol. Neurobiol.* 38 995–1007. 10.1007/s10571-018-0584-7 29687234 PMC11481975

[B13] Cohen J. Torres C. (2019). Astrocyte senescence: evidence and significance. *Aging Cell* 18:e12937. 10.1111/acel.12937 30815970 PMC6516680

[B14] Cohen J. D’Agostino L. Tuzer F. Torres C. (2018). HIV antiretroviral therapy drugs induce premature senescence and altered physiology in HUVECs. *Mech. Ageing Dev.* 175 74–82. 10.1016/j.mad.2018.07.008 30055190 PMC6133242

[B15] Cohen J. D’Agostino L. Wilson J. Tuzer F. Torres C. (2017). Astrocyte senescence and metabolic changes in response to HIV antiretroviral therapy drugs. *Front. Aging Neurosci.* 9:281. 10.3389/fnagi.2017.00281 28900395 PMC5581874

[B16] Dienel G. A. (2017). The metabolic trinity, glucose-glycogen-lactate, links astrocytes and neurons in brain energetics, signaling, memory, and gene expression. *Neurosci. Lett.* 637 18–25. 10.1016/j.neulet.2015.02.052 25725168

[B17] Dimri G. P. Lee X. Basile G. Acosta M. Scott G. Roskelley C. (1995). A biomarker that identifies senescent human cells in culture and in aging skin in vivo. *Proc. Natl. Acad. Sci. U.S.A.* 92 9363–9367. 10.1073/pnas.92.20.9363 7568133 PMC40985

[B18] Dringen R. Gutterer J. M. Hirrlinger J. (2000). Glutathione metabolism in brain metabolic interaction between astrocytes and neurons in the defense against reactive oxygen species. *Eur. J. Biochem.* 267 4912–4916. 10.1046/j.1432-1327.2000.01597.x 10931173

[B19] Franceschi C. Capri M. Monti D. Giunta S. Olivieri F. Sevini F. (2007). Inflammaging and anti-inflammaging: a systemic perspective on aging and longevity emerged from studies in humans. *Mech. Ageing Dev.* 128 92–105. 10.1016/j.mad.2006.11.016 17116321

[B20] Gerónimo-Olvera C. Montiel T. Rincon-Heredia R. Castro-Obregón S. Massieu L. (2017). Autophagy fails to prevent glucose deprivation/glucose reintroduction-induced neuronal death due to calpain-mediated lysosomal dysfunction in cortical neurons. *Cell Death Dis.* 8:e2911. 10.1038/cddis.2017.299 28661473 PMC5520945

[B21] Guerrero A. De Strooper B. Arancibia-Cárcamo I. L. (2021). Cellular senescence at the crossroads of inflammation and Alzheimer’s disease. *Trends Neurosci.* 44 714–727. 10.1016/j.tins.2021.06.007 34366147

[B22] Hernandez-Segura A. Nehme J. Demaria M. (2018). Hallmarks of cellular senescence. *Trends Cell Biol.* 28 436–453. 10.1016/j.tcb.2018.02.001 29477613

[B23] Jha M. K. Morrison B. M. (2018). Glia-neuron energy metabolism in health and diseases: new insights into the role of nervous system metabolic transporters. *Exp. Neurol.* 309 23–31. 10.1016/j.expneurol.2018.07.009 30044944 PMC6156776

[B24] Jiang T. Cadenas E. (2014). Astrocytic metabolic and inflammatory changes as a function of age. *Aging Cell* 13 1059–1067. 10.1111/acel.12268 25233945 PMC4244278

[B25] Kirchhoff F. Dringen R. Giaume C. (2001). Pathways of neuron-astrocyte interactions and their possible role in neuroprotection. *Eur. Arch. Psychiatry Clin. Neurosci.* 251 159–169. 10.1007/s004060170036 11697580

[B26] LaFrance R. Brustovetsky N. Sherburne C. Delong D. Dubinsky J. M. (2005). Age-related changes in regional brain mitochondria from Fischer 344 rats. *Aging Cell* 4 139–145. 10.1111/j.1474-9726.2005.00156.x 15924570

[B27] Limbad C. Oron T. R. Alimirah F. Davalos A. R. Tracy T. E. Gan L. (2020). Astrocyte senescence promotes glutamate toxicity in cortical neurons. *PLoS One* 15:e0227887. 10.1371/journal.pone.0227887 31945125 PMC6964973

[B28] López-Otín C. Blasco M. A. Partridge L. Serrano M. Kroemer G. (2013). The hallmarks of aging. *Cell* 153 1194–1217. 23746838 10.1016/j.cell.2013.05.039 PMC3836174

[B29] Maciel-Barón L. Á Moreno-Blas D. Morales-Rosales S. L. González-Puertos V. Y. López-Díazguerrero N. E. Torres C. (2018b). Cellular senescence, neurological function, and redox state. *Antioxid. Redox Signal.* 28 1704–1723. 10.1089/ars.2017.7112 28467755

[B30] Maciel-Barón L. Á Morales-Rosales S. L. Silva-Palacios A. Rodríguez-Barrera R. H. García-Álvarez J. A. Luna-López A. (2018a). The secretory phenotype of senescent astrocytes isolated from Wistar newborn rats changes with anti-inflammatory drugs, but does not have a short-term effect on neuronal mitochondrial potential. *Biogerontology* 19 415–433. 10.1007/s10522-018-9767-3 30097900

[B31] Matias I. Morgado J. Gomes F. C. A. (2019). Astrocyte heterogeneity: impact to brain aging and disease. *Front. Aging Neurosci.* 11:59. 10.3389/fnagi.2019.00059 30941031 PMC6433753

[B32] Mattson M. P. Arumugam T. V. (2018). Hallmarks of brain aging: adaptive and pathological modification by metabolic states. *Cell Metab.* 27 1176–1199. 10.1016/j.cmet.2018.05.011 29874566 PMC6039826

[B33] Mattson M. P. Gleichmann M. Cheng A. (2008). Mitochondria in neuroplasticity and neurological disorders. *Neuron* 60 748–766. 10.1016/j.neuron.2008.10.010 19081372 PMC2692277

[B34] McCarthy K. D. de Vellis J. (1980). Preparation of separate astroglial and oligodendroglial cell cultures from rat cerebral tissue. *J. Cell Biol.* 85 890–902. 10.1083/jcb.85.3.890 6248568 PMC2111442

[B35] Merrill R. A. Flippo K. H. Strack S. (2017). “Measuring mitochondrial shape with imageJ,” in *Techniques to Investigate Mitochondrial Function in Neurons*, eds Strack S. Usachev Y. M. (New York, NY: Springer New York), 31–48. 10.1007/978-1-4939-6890-9_2

[B36] Missiroli S. Genovese I. Perrone M. Vezzani B. Vitto V. A. M. Giorgi C. (2020). The role of mitochondria in inflammation: from cancer to neurodegenerative disorders. *J. Clin. Med. Res.* 9:740. 10.3390/jcm9030740 32182899 PMC7141240

[B37] Nakamura T. Cieplak P. Cho D.-H. Godzik A. Lipton S. A. (2010). S-nitrosylation of Drp1 links excessive mitochondrial fission to neuronal injury in neurodegeneration. *Mitochondrion* 10 573–578. 10.1016/j.mito.2010.04.007 20447471 PMC2918703

[B38] Pannese E. (2021). Quantitative, structural and molecular changes in neuroglia of aging mammals: a review. *Eur. J. Histochem.* 65:3249. 10.4081/ejh.2021.3249 34346664 PMC8239453

[B39] Perrott K. M. Wiley C. D. Desprez P.-Y. Campisi J. (2017). Apigenin suppresses the senescence-associated secretory phenotype and paracrine effects on breast cancer cells. *Geroscience* 39 161–173. 10.1007/s11357-017-9970-1 28378188 PMC5411372

[B40] Posadas-Rodríguez P. Posadas-Rodríguez N. E. González-Puertos V. Y. Toledo-Pérez R. Ventura-Gallegos J. L. Zentella A. (2020). tBHQ induces a hormetic response that protects L6 myoblasts against the toxic effect of palmitate. *Oxid. Med. Cell. Longev.* 2020:3123268. 10.1155/2020/3123268 32509140 PMC7246405

[B41] Ricci G. Volpi L. Pasquali L. Petrozzi L. Siciliano G. (2009). Astrocyte-neuron interactions in neurological disorders. *J. Biol. Phys.* 35 317–336. 10.1007/s10867-009-9157-9 19669420 PMC2750745

[B42] Ritschka B. Storer M. Mas A. Heinzmann F. Ortells M. C. Morton J. P. (2017). The senescence-associated secretory phenotype induces cellular plasticity and tissue regeneration. *Genes Dev.* 31 172–183. 10.1101/gad.290635.116 28143833 PMC5322731

[B43] Rivero-Segura N. A. Coronado-Mares M. I. Rincón-Heredia R. Pérez-Torres I. Montiel T. Pavón N. (2019). Prolactin prevents mitochondrial dysfunction induced by glutamate excitotoxicity in hippocampal neurons. *Neurosci. Lett.* 701 58–64. 10.1016/j.neulet.2019.02.027 30790645

[B44] Rodier F. Campisi J. (2011). Four faces of cellular senescence. *J. Cell Biol.* 192 547–556. 10.1083/jcb.201009094 21321098 PMC3044123

[B45] Takahashi S. (2021). Neuroprotective function of high glycolytic activity in astrocytes: common roles in stroke and neurodegenerative diseases. *Int. J. Mol. Sci.* 22:6568. 10.3390/ijms22126568 34207355 PMC8234992

[B46] Theurey P. Pizzo P. (2018). The aging mitochondria. *Genes* 9:22. 10.3390/genes9010022 29315229 PMC5793175

[B47] Triana-Martínez F. Picallos-Rabina P. Da Silva-Álvarez S. Pietrocola F. Llanos S. Rodilla V. (2019). Identification and characterization of Cardiac Glycosides as senolytic compounds. *Nat. Commun.* 10:4731. 31636264 10.1038/s41467-019-12888-x PMC6803708

[B48] Turnquist C. Horikawa I. Foran E. Major E. O. Vojtesek B. Lane D. P. (2016). p53 isoforms regulate astrocyte-mediated neuroprotection and neurodegeneration. *Cell Death Differ.* 23 1515–1528. 10.1038/cdd.2016.37 27104929 PMC5072428

[B49] Van Eldik L. J. Carrillo M. C. Cole P. E. Feuerbach D. Greenberg B. D. Hendrix J. A. (2016). The roles of inflammation and immune mechanisms in Alzheimer’s disease. *Alzheimers. Dement.* 2 99–109. 10.1016/j.trci.2016.05.001 29067297 PMC5644267

[B50] Vicente-Gutiérrez C. Jiménez-Blasco D. Quintana-Cabrera R. (2021). Intertwined ROS and metabolic signaling at the neuron-astrocyte interface. *Neurochem. Res.* 46 23–33. 10.1007/s11064-020-02965-9 31989468

[B51] Wiley C. D. Campisi J. (2016). From Ancient pathways to aging cells-connecting metabolism and cellular senescence. *Cell Metab.* 23 1013–1021. 10.1016/j.cmet.2016.05.010 27304503 PMC4911819

[B52] Yan J. Fu Q. Cheng L. Zhai M. Wu W. Huang L. (2014). Inflammatory response in Parkinson’s disease (Review). *Mol. Med. Rep.* 10 2223–2233. 25215472 10.3892/mmr.2014.2563

