# Supplementary figures and images for: Senescence in Primary Rat Astrocytes Induces Loss of the Mitochondrial Membrane Potential and Alters Mitochondrial Dynamics in Cortical Neurons
Source: Front Aging Neurosci. 2021 Dec 1;13:766306. doi: 10.3389/fnagi.2021.766306 (PMC8672143; doi:10.3389/fnagi.2021.766306)

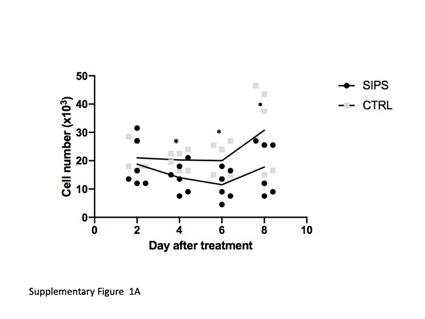

Supplement: Supplementary Figure 1 — (A) Astrocytes proliferation. Cellular proliferation was determined every other day in hydrogen peroxide (H2O2) treated and untreated astrocytes. Each point represents the mean ± SD of three independent experiments. Statistical significance tested by the ANOVA and Tukey–Kramer test ∗p ≤ 0.05 with respect to control cells. (B) Senescence-associated-β-galactosidase (SA-β-Gal) staining. Representative images of H2O2 treated and untreated astrocytes. (C) SA-β-Gal-positive cells quantification. Each point represents the mean ± SD of three independent experiments. Statistical significance tested by the ANOVA and the Tukey–Kramer test ∗p ≤ 0.05 with respect to control cells. [file Image_1.JPEG]

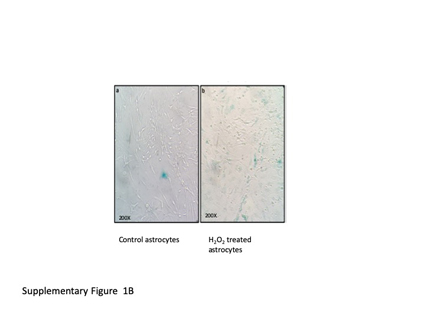

Supplement: Supplementary Figure 2 — Mitochondrial morphology was evaluated by fluorescent microscopy by using MitoTracker red as described in the methodology. (A) Representative images of the three basic morphologies used for classification: tubular, fragmented, and intermediate. (B) Representative images observed in neurons, neurons +CA, and neurons +SA. (C) The percentage of evaluated morphologies in each condition. A total of 50 cells were blindly counted per treatment. [file Image_2.JPEG]

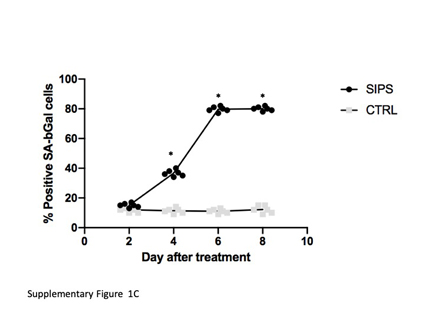

Supplement: Supplementary file 3 [file Image_3.JPEG]

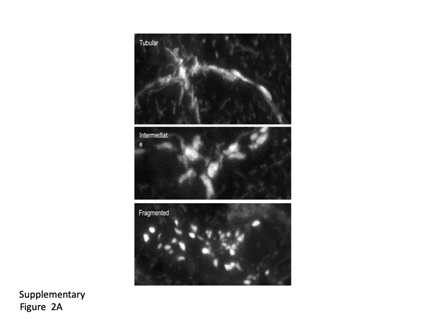

Supplement: Supplementary file 4 [file Image_4.JPEG]

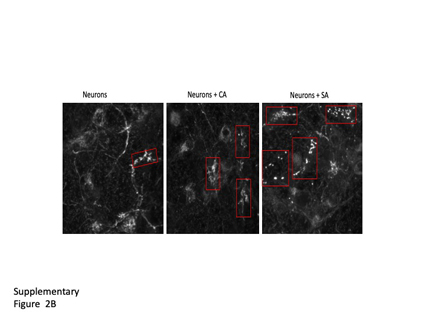

Supplement: Supplementary file 5 [file Image_5.JPEG]

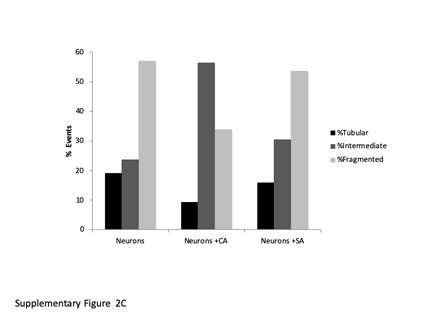

Supplement: Supplementary file 6 [file Image_6.JPEG]
